# Buried electrostatic modulation enables size-dependent reactivity in Pd-based nanocatalysts

**DOI:** 10.1039/d5sc06015j

**Published:** 2025-11-26

**Authors:** Tzu-An Chou, Hsiang-Yu Yu, Hui-Yun Lo, Yu-Ting Chen, Zhi-Wei Wang, Hsin-Lun Wu

**Affiliations:** a Department of Chemistry, National Cheng Kung University Tainan 70101 Taiwan wuhsinlun@mail.ncku.edu.tw

## Abstract

The catalytic reactivity of metal nanocatalysts is generally assumed to plateau beyond tens of nanometers as their electronic structure and Fermi level (*E*_F_) converge with bulk properties. Surprisingly, our catalytic experiments reveal that Au nanocubes retain pronounced and switchable size-dependent reactivity even above 30 nm, while Pd nanocubes remain size-invariant in this regime. This intriguing contrast parallels their plasmonic behavior, where Au's sp-electron dominance enables robust plasmonic activity despite Pd's strong d-electron damping. Crucially, unlike prior Au–Pd studies focused on static d-band shifts (ligand effects) or light-induced hot electron effects (plasmonics), we propose that Au's sp-electron dominance enables strong *E*_F_ responsiveness to transient surface charge, whereas Pd's d-states near *E*_F_ likely buffer these electrostatic perturbations. Leveraging this fundamental contrast, we designed Au–Pd core–shell nanocubes where the buried Au core serves as an internal electrostatic modulator. By sensing and amplifying surface charge, the Au core dynamically tunes Pd shell reactivity, enabling reversible and size-dependent modulation under reductive *versus* oxidative conditions—a behavior unattainable with Pd alone and unprecedented in this large size regime. This rational design introduces a new electrostatic degree of freedom in nanocatalysis, establishing *E*_F_ responsiveness—not d-band energetics or surface coordination—as a foundational principle for charge- and size-tunable catalysis.

## Abstract 2

Electrostatic responsiveness is introduced as a new design principle for nanocatalysis, showing how buried Au cores can amplify charge effects to reversibly tune Pd shell reactivity with particle size.

## Graphical abstract

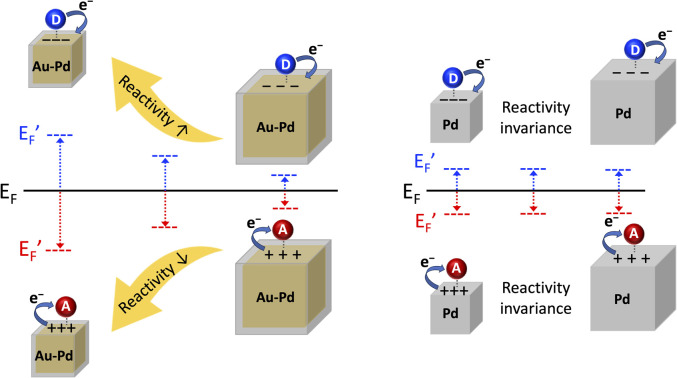

## Introduction

The catalytic reactivity of transition metal nanoparticles is fundamentally dictated by their surface electronic states, which govern interactions with adsorbates. Traditional bonding models, emphasizing localized d-states, largely attribute variations in reactivity to shifts in d-band characteristics, with the role of broader, delocalized sp-states often assumed to be secondary.^1–7^ This framework generally predicts that catalytic properties, including size effects, should plateau beyond tens of nanometers as nanoparticles increasingly resemble their bulk counterparts and their electronic structures, including the Fermi level (*E*_F_), converge with bulk properties.^8–10^

**Fig. 1:**
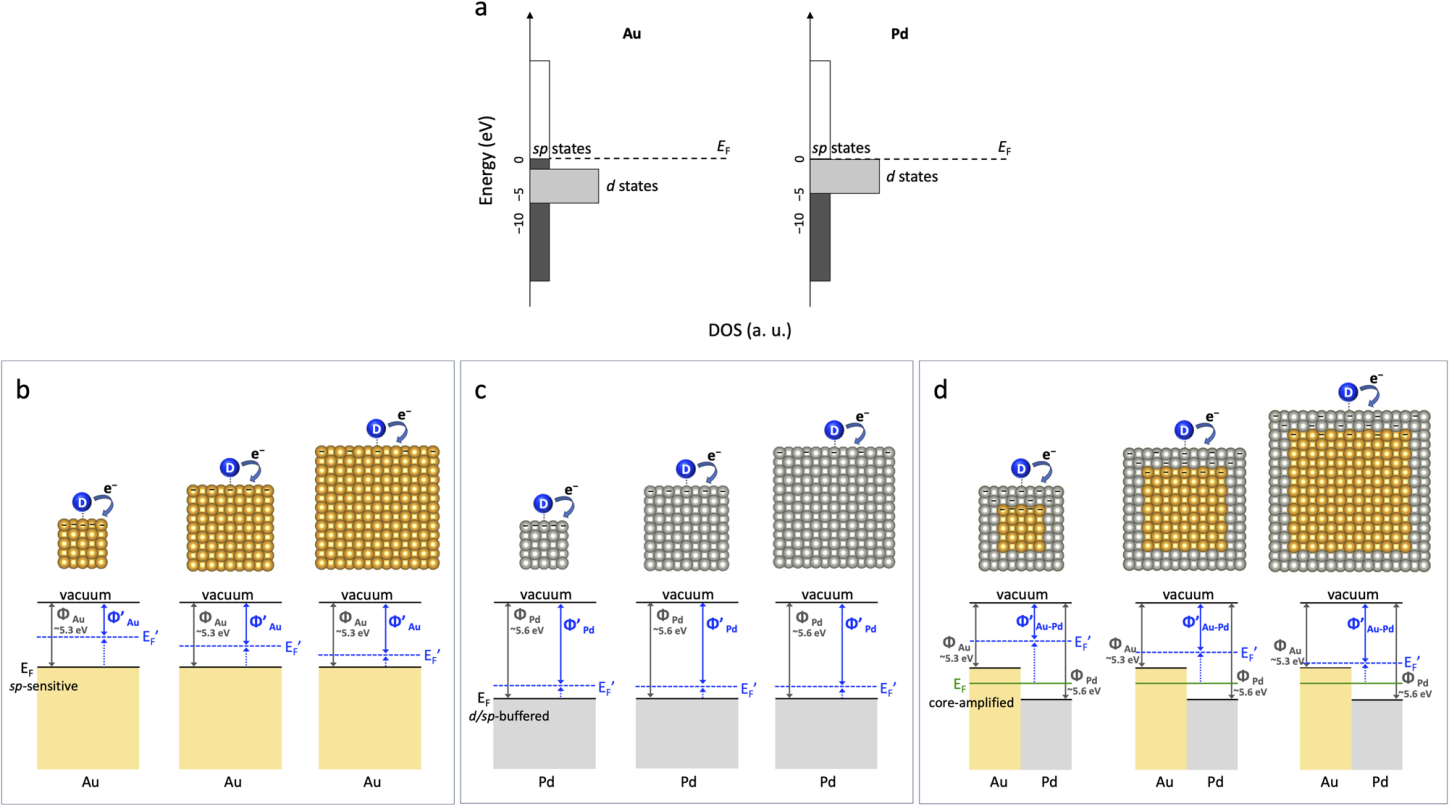
Schematic illustration of electronic structure and electrostatic responsiveness in nanocatalysts. (a) Density of state (DOS) for Au and Pd, highlighting distinct sp- and d-state contributions near the Fermi level (*E*_F_). (b) Au nanocubes show pronounced *E*_F_ elevation under transient negative surface charge accumulation, with stronger effects at smaller sizes. (c) Pd nanocubes exhibit buffered *E*_F_ shifts with weak size dependence, reflecting their low electrostatic responsiveness. (d) In Au–Pd core–shell nanocubes, electron flow from Au (work function *Φ*_Au_: 5.3 eV) to Pd (*Φ*_Pd_: 5.6 eV) establishes an equilibrium *E*_F_ (green line) representing the ligand effect, while the buried Au core further amplifies *E*_F_ elevation induced by transient negative surface charge accumulation, especially in smaller particles.

Surprisingly, our catalytic experiments reveal that Au nanocubes retain pronounced and switchable size-dependent reactivity even above 30 nm, a regime where traditional models would predict bulk-like behavior. In stark contrast, Pd nanocubes remain size-invariant under the same conditions. This intriguing divergence in catalytic behavior parallels their well-known plasmonic properties: while both Au and Pd support plasmonic excitations, Pd's strong d-electron contributions dampen optical resonance, whereas Au's sp-electron dominance enables robust plasmonic activity.^11,12^ Crucially, while previous work on Au–Pd systems has explored static d-band shifts (ligand effects)^13–17^ and light-induced hot electron effects (plasmonics),^12,18–21^ the direct role of this intrinsic sp-electron dominance in governing electrostatic effects on catalysis—particularly under dark conditions—has remained elusive. As schematically illustrated in Fig. 1, we hypothesize that this intrinsic electronic structure difference dictates the *E*_F_ responsiveness to transient electron redistribution at the catalyst surface during reactions. Under reductive conditions, such electron accumulation can raise *E*_F_ to enhance reactivity,^22,23^ whereas under oxidative conditions, electron depletion can lower *E*_F_ to suppress it, thereby governing size-dependent catalytic activity. Importantly, this “accumulation/depletion” refers to dynamic shifts in electronic structure rather than the net colloidal charge typically probed by zeta potential measurements.

Building upon this hypothesis, we explored how the distinct *E*_F_ responsiveness of Au and Pd influences their catalytic behavior across varying sizes and transient surface charge environments. We then rationally designed Au–Pd core–shell nanocubes to leverage Au's unique electrostatic sensitivity, aiming to modulate Pd's catalytic activity. Our findings demonstrate that a buried Au core can indeed serve as an internal electrostatic modulator, enabling dynamic, switchable, and size-dependent tuning of Pd shell reactivity, even when the system approaches bulk-like dimensions—effects unattainable with monometallic Pd. This work ultimately establishes *E*_F_ responsiveness, amplified by core–shell synergy, as a central and foundational principle for designing charge- and size-tunable nanocatalysts, advancing the field beyond static d-band and light-induced plasmonic mechanisms.

## Results and discussion

**Fig. 2:**
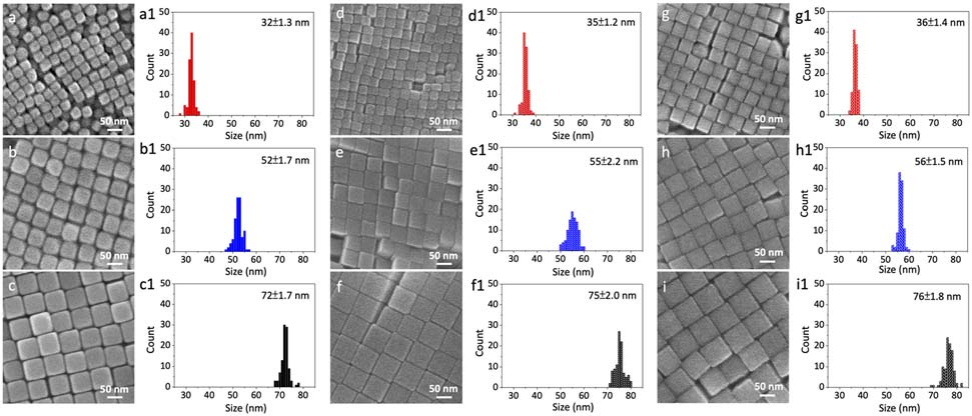
Morphological characterization of synthesized nanocubes. SEM images of (a–c) Au, (d–f) Pd, and (g–i) Au–Pd core–shell nanocubes, with corresponding size distributions shown in (a1–c1), (d1–f1), and (g1–i1), respectively.

To experimentally probe the proposed interplay between intrinsic electronic structure and *E*_F_ responsiveness in nanocatalysis, we synthesized Au, Pd, and Au–Pd core–shell nanocubes in three well-defined sizes (∼35, 55, and 75 nm) using a seed-mediated growth method.^24–27^ Scanning electron microscopy (SEM) images (Fig. 2a–i) confirmed their uniform cubic morphology and narrow size distributions (Fig. 2a1–i1). Specifically, the Au nanocubes measured 32 ± 1.3, 52 ± 1.7, and 72 ± 1.7 nm; the Pd nanocubes were 35 ± 1.2, 55 ± 2.2, and 75 ± 2.0 nm; and the Au–Pd core–shell nanocubes, formed by overgrowing a ∼2 nm thin Pd shell on Au cores, were 36 ± 1.4, 56 ± 1.5, and 76 ± 1.8 nm.

To further examine the effect of shell thickness on electronic coupling and electrostatic responsiveness, as well as to assess the potential influence of lattice strain, we also prepared a set of Au–Pd core–shell nanocubes with a thicker (∼12 nm) Pd shell (denoted as “thick-shell” Au–Pd). SEM images and size distributions of the thick-shell (∼12 nm) Au–Pd core–shell nanocubes are shown in Fig. S1. High-angle annular dark-field scanning transmission electron microscopy (HAADF-STEM) and energy-dispersive X-ray spectroscopy (EDS) mapping unequivocally confirmed the core–shell structure with uniform Pd shell encapsulating the Au cores for both thin- and thick-shell samples (Fig. S2), while X-ray diffraction (XRD) patterns verified their corresponding crystal structures (Fig. S3).

These precisely size-controlled nanocubes served as an ideal platform for systematically investigating how reaction-induced transient surface charge density, which inherently varies with particle size and shell thickness, influences *E*_F_ dynamics and, consequently, catalytic reactivity. This rigorous approach directly allowed us to test our hypothesis regarding Au's electrostatic sensitivity from its sp-state contributions *versus* Pd's relative d/sp-state stability, and the critical role of the Au core in tuning the Pd shell's catalytic behavior.

**Fig. 3:**
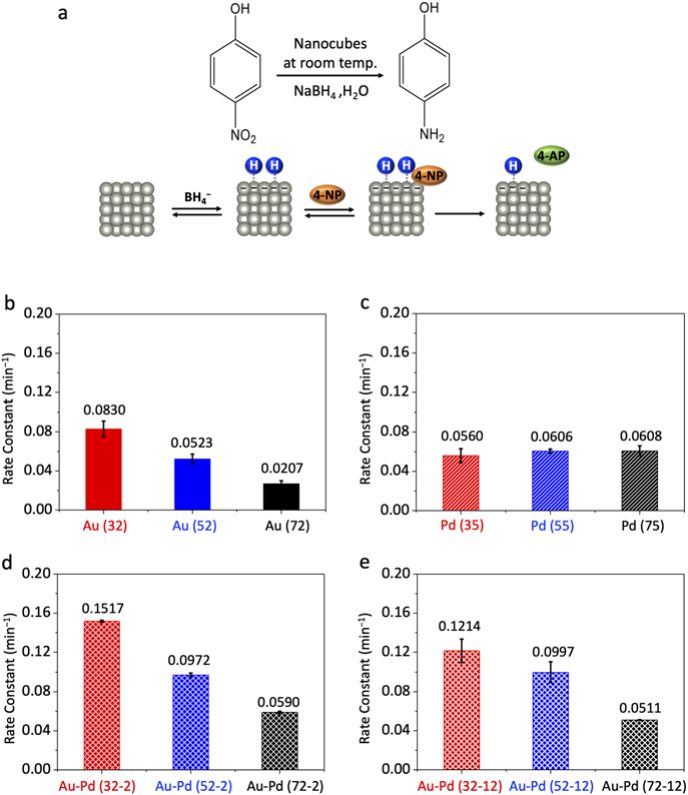
Catalytic performance of nanocubes in 4-nitrophenol reduction. (a) Schematic illustration of the reaction mechanism, emphasizing how borohydride adsorption generates transient negative surface charge accumulation on the catalyst surface, which modulates the effective *E*_F_ and thereby influences catalytic activity. Size-dependent reaction rate constants for: (b) Au nanocubes; (c) Pd nanocubes; (d) thin-shell Au–Pd core–shell nanocubes; and (e) thick-shell Au–Pd core–shell nanocubes.

We first evaluated the catalytic performance of Au, Pd, and Au–Pd core–shell nanocubes in the reduction of 4-nitrophenol, a model reaction known to generate a transiently negatively charged surface upon borohydride adsorption (Fig. 3a). The conversion of 4-nitrophenol to 4-aminophenol in the presence of excess NaBH_4_ followed pseudo-first-order kinetics.^28–32^ UV-vis spectroscopy (Fig. S4) showed a diminishing absorption peak at ∼400 nm (4-nitrophenol consumption) and a simultaneous increase at ∼300 nm (4-aminophenol formation). We extracted rate constants by monitoring the 400 nm absorbance over time and plotting ln(*A*/*A*_0_) *versus* time (Fig. S5).

For Au nanocubes, we observed a pronounced size-dependent increase in reactivity (Fig. 3b). Specifically, the rate constants for 32, 52, and 72 nm Au nanocubes were 0.0830 ± 0.0080, 0.0523 ± 0.0050, and 0.0207 ± 0.0030 min^−1^, respectively. This trend aligns with our hypothesis that smaller Au nanoparticles, experiencing more pronounced transient electron redistribution and higher local negative surface charge density during the reaction, exhibit a more significantly elevated *E*_F_, thereby enhancing reactivity. In stark contrast, Pd nanocubes (35, 55, and 75 nm) showed negligible size dependence, with rate constants of 0.0560 ± 0.0070, 0.0606 ± 0.0020, and 0.0608 ± 0.0050 min^−1^ (Fig. 3c). This size-invariant trend for Pd is consistent with its intrinsic electrostatic inertness, where d/sp-state overlap near the *E*_F_ effectively dampens the influence of transient surface charge modulation.

Remarkably, thin-shell Au–Pd core–shell nanocubes (36, 56, and 76 nm) displayed pronounced size-dependent reactivity in 4-nitrophenol reduction, with rate constants of 0.1517 ± 0.0010, 0.0972 ± 0.0020, and 0.0590 ± 0.0010 min^−1^ (Fig. 3d). To unequivocally disentangle electrostatic modulation from lattice strain effects, we evaluated thick-shell (∼12 nm) Au–Pd core–shell nanocubes. As shown in Fig. 3e, these thick-shell nanocubes continue to exhibit a size-dependent trend in catalytic activity (0.1214 ± 0.0117, 0.0997 ± 0.0105, 0.0511 ± 0.0003 min^−1^ from small to large), despite the shell thickness far exceeding the typical critical thickness for strain dissipation (∼3 to 5 nm). This observation confirms that the buried Au core still modulates the Pd shell *via* electrostatic effects, although the overall activity is reduced compared to thin-shell samples due to partial charge screening in the thicker shell. The persistent size-dependent behavior, coupled with the negligible variation in lattice strain among these large sizes, definitively establishes electrostatic responsiveness as the dominant factor governing reactivity in the core–shell system.

Notably, this size-dependent trend for Au–Pd core–shell nanocubes mirrored that of pure Au, where smaller particles exhibited higher reactivity. This finding suggests that the buried Au core effectively imparts its electrostatic responsiveness to the Pd shell, thereby influencing its catalytic behavior. Furthermore, Au–Pd core–shell nanocubes generally outperformed both monometallic counterparts in this reaction, indicating a synergistic effect between the two metals arising from combined ligand and electrostatic effects.

Unlike bimetallic Janus nanocrystals, where two metals are simultaneously exposed and the interface directly participates in charge transfer and molecular activation,^33,34^ the Au–Pd nanocubes studied here possess a core–shell configuration in which the Au core is completely encapsulated by the Pd shell. Consequently, the catalytic surface consists exclusively of Pd atoms, and the Au–Pd interface does not directly interact with reactants. The observed synergistic enhancement thus originates from electronic modulation of the Pd shell induced by the buried Au core. Specifically, electron flow from Au (work function 5.3 eV) to Pd (5.6 eV) establishes an equilibrium Fermi level (ligand effect) that elevates the Pd *E*_F_,^13–17^ facilitating electron donation to the nitro group (NO_2_^−^) during the rate-determining step of 4-nitrophenol reduction and thereby enhancing catalytic activity.^28,29^

**Fig. 4:**
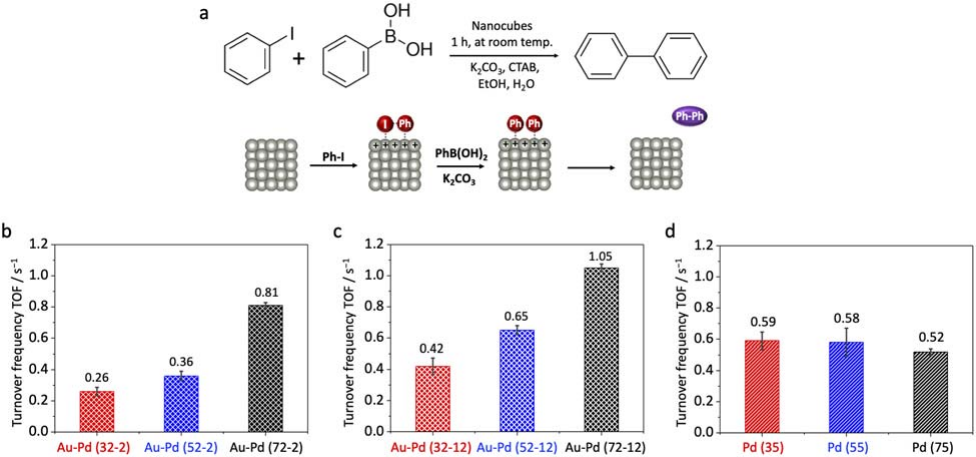
Catalytic performance of nanocubes in Suzuki coupling reaction. (a) Schematic illustration of the reaction mechanism, emphasizing how oxidative addition of iodobenzene under basic conditions induces transient positive surface charge accumulation on the catalyst surface, which modulates the effective *E*_F_ and thus affects catalytic activity. Size-dependent turnover frequencies (TOFs) for: (b) thin-shell Au–Pd core–shell nanocubes; (c) thick-shell Au–Pd core–shell nanocubes; and (d) Pd nanocubes.

To further investigate the electrostatic modulation of catalytic activity under varying charge polarities, we next evaluated the performance of Au–Pd core–shell and Pd nanocubes in the Suzuki coupling reaction (Fig. 4a). This reaction, involving the oxidative addition of iodobenzene onto the Pd surface in a basic medium, is well-established to generate a transiently positively charged surface.^35–39^ Given Pd's critical role in this reaction, and to directly probe the Au core's influence in this electron-deficient environment, our focus was exclusively on these Au–Pd core–shell and monometallic Pd nanocubes. GC-MS analysis confirmed that biphenyl was the only coupling product detected, with no observable side products. Control reactions using only iodobenzene or phenylboronic acid yielded no biphenyl, confirming nearly 100% selectivity of the Suzuki coupling under the applied conditions (Fig. S6).

In striking contrast, thin-shell Au–Pd core–shell nanocubes (36, 56, and 76 nm) displayed a clear size-dependent reactivity in Suzuki coupling (Fig. 4b). Their turnover frequencies (TOFs) were 0.26 ± 0.027, 0.36 ± 0.030, and 0.81 ± 0.018 s^−1^, respectively. Notably, the trend observed for Au–Pd core–shell nanocubes in Suzuki coupling was opposite to that in 4-nitrophenol reduction: smaller particles exhibited suppressed reactivity, while larger particles showed enhanced activity. This striking divergence underscores the ability of the Au core's sp-state sensitivity to transient surface charge to induce switchable size-dependent trends. In this transiently positively charged environment, the *E*_F_ is inferred to lower, suppressing reactivity, with this effect being more pronounced at smaller sizes due to greater transient positive charge accumulation (Fig. S7).

To further confirm that the observed size-dependent trends originate from electrostatic modulation rather than lattice strain, we also examined thick-shell (∼12 nm) Au–Pd core–shell nanocubes. These thick-shell nanocubes exhibited TOFs of 0.42 ± 0.053, 0.65 ± 0.029, and 1.05 ± 0.025 s^−1^ from small to large (Fig. 4c). Consistent with the findings from 4-nitrophenol reduction, the thick-shell structures retained the size-dependent trend observed in the thin-shell samples, with smaller particles showing lower reactivity. As previously established (Fig. 3e), this confirms that electrostatic responsiveness, rather than lattice strain, governs the size-dependent behavior in the Au–Pd system. Furthermore, TEM characterization after both 4-nitrophenol reduction and Suzuki coupling reactions confirmed that both thin- and thick-shell Au–Pd core–shell nanocubes maintained their cubic morphology and well-defined core–shell structures, indicating that the catalysts are structurally stable under the applied reaction conditions (Fig. S8). This structural stability further supports that the observed size-dependent catalytic trends are primarily governed by charge-sensitive modulation from the buried Au core, rather than by morphological changes, alloying, or metal leaching.

Compared to thin-shell samples, the overall catalytic activity of thick-shell nanocubes was slightly higher, despite retaining the same size-dependent trend. This difference arises from partial screening of the buried Au core's electrostatic influence: the Au core establishes an equilibrium *E*_F_*via* electron transfer to the Pd shell (ligand effect), and transient surface charge accumulation during the reaction (electrostatic effect) further modulates this *E*_F_. In thick-shell nanocubes, the electrostatic coupling from the Au core is weakened, so the *E*_F_ shift induced by the positively charged reaction environment is smaller than in thin-shell samples. Consequently, the activity suppression due to *E*_F_ lowering is partially mitigated, while the ligand-established *E*_F_ baseline remains.

This observation highlights the competing influences of ligand and electrostatic effects: increasing the shell thickness diminishes the electrostatic contribution without eliminating the underlying size-dependent behavior. The results demonstrate that the buried Au core primarily governs Pd-shell reactivity *via* charge-sensitive modulation, and that the strength of this electrostatic coupling can be tuned by shell thickness in a charge-polarity-dependent manner.

For Pd nanocubes (35, 55, and 75 nm), TOFs were 0.59 ± 0.055, 0.58 ± 0.090, and 0.52 ± 0.020 s^−1^, respectively (Fig. 4d). Consistent with our observations in 4-nitrophenol reduction, Pd nanocubes again showed a negligible size dependence in Suzuki coupling, thereby reinforcing their intrinsic electrostatic inertness across different reaction environments and charge polarities.

Our findings demonstrate that whether a nanocatalyst exhibits size-dependent reactivity is governed not by the static position of the *E*_F_, but by its intrinsic electrostatic responsiveness—that is, the capacity of its *E*_F_ to undergo significant shifts in response to transient surface charge accumulation or depletion. This *E*_F_ shift, which is naturally enhanced in smaller particles due to higher transient surface charge density, determines the extent of catalytic modulation across different surface charge environments.

The observed distinct catalytic behaviors of Au and Pd directly reflect their underlying electronic structures and corresponding *E*_F_ responsiveness. Au's *E*_F_, dominated by highly delocalized sp-states, exhibits strong shifts in response to transient surface charge. For instance, under negatively charged conditions (as in 4-nitrophenol reduction), *E*_F_ is elevated. This *E*_F_ elevation is more pronounced at smaller Au particle sizes due to greater transient negative charge accumulation, leading to the observed size-dependent enhancement in reactivity. Conversely, under positively charged conditions (as in Suzuki coupling for Au–Pd system), *E*_F_ is lowered. This *E*_F_ suppression is also more significant at smaller particle sizes, causing a suppression of reactivity.

In stark contrast, Pd's *E*_F_, characterized by overlapping d- and sp-states, exhibits a buffered electrostatic response. The d/sp orbital overlap near the *E*_F_ effectively dampens the influence of transient surface charge modulation, leading to its consistent size-invariant *E*_F_ shifts and catalytic responses under both negative and positive charge polarities. This fundamental difference explains why Pd nanocubes show negligible size dependence in both 4-nitrophenol reduction and Suzuki coupling, despite variations in surface charge environment with size.

Crucially, Au–Pd core–shell nanocubes harness the charge-sensitive nature of the buried Au core to actively modulate the Pd shell's reactivity. Initially, the Au (5.3 eV) to Pd (5.6 eV) electron flow establishes an equilibrium *E*_F_ (the baseline ligand effect) that enriches the Pd shell. Beyond this static state, the Au core acts as an internal electrostatic amplifier, mediating *E*_F_ shifts in the Pd shell based on surface charge polarity and particle size. Under negatively charged conditions (4-nitrophenol reduction), the Au core amplifies transient *E*_F_ elevation in the Pd shell, enhancing reactivity; conversely, under positively charged conditions (Suzuki coupling), it amplifies transient *E*_F_ lowering, suppressing reactivity. This switchable, dual behavior leads to opposite size-dependent trends unobserved for Pd alone. Ultimately, while the ligand effect sets the equilibrium *E*_F_, transient electrostatic modulation dominates in smaller Au–Pd nanocubes due to higher transient surface charge density, amplifying the enhancement or suppression of catalytic activity by surface charge. In larger Au–Pd nanocubes, the weaker transient *E*_F_ shifts result in less pronounced modulation of activity by surface charge and the ligand effect dominates.

These findings underscore that size-dependent catalytic performance in core–shell nanocatalysts arises primarily from transient electrostatic modulation of the *E*_F_, mediated by the charge-responsive Au core. This mechanism departs from traditional d-state-centered models and highlights the transient surface charge polarity generated during the catalytic reaction, in combination with nanoscale dimensions and the intrinsic *E*_F_ responsiveness of the catalyst material, as decisive factors governing catalytic behavior.

## Conclusions

Our study demonstrates that the intrinsic electrostatic responsiveness of metal nanocatalysts—governed by their *E*_F_ electronic structure—dictates whether they exhibit size-dependent catalytic behavior. Au nanocubes, with *E*_F_ dominated by highly charge-sensitive sp-states, display pronounced reactivity modulation with particle size under varying transient surface charge conditions, reflecting strong susceptibility to electrostatic perturbations. In contrast, Pd nanocubes, characterized by overlapping d- and sp-states near *E*_F_, exhibit size-invariant reactivity, suggesting that such electronic configurations buffer against charge-induced modulation. This distinct divergence is consistently observed across both electron-rich (4-nitrophenol reduction) and electron-deficient (Suzuki coupling) environments. Leveraging this contrast, we designed Au–Pd core–shell nanocubes in which the buried Au core acts as an internal electrostatic amplifier. By sensing and amplifying transient surface charge, the Au core enables reversible, size- and charge-dependent tuning of Pd shell reactivity—an effect unattainable in Pd alone. These findings reveal that size-dependent catalytic behavior arises not from traditional d-band or coordination models, but from how sensitively *E*_F_ responds to surface charge perturbations. This insight offers a unifying framework for understanding divergent size effects in nanocatalysis and provides a powerful design principle for developing charge-responsive nanocatalysts through controlled tuning of size, composition, and electronic structure.

While the present discussion of *E*_F_ responsiveness is qualitative and based on experimental observations, future computational modeling and direct spectroscopic studies (*e.g.*, ultrafast transient absorption, *in situ* XPS, or *operando* studies) will be valuable for quantitatively establishing the relationship between *E*_F_ modulation and catalytic activity. Looking forward, we aim to extend this concept to more mechanistically complex catalytic systems—such as CO_2_ reduction, selective hydrogenation, and oxygen evolution—where charge-polarized environments may play an even more decisive role in determining activity and selectivity.

## Author contributions

T.-A. C., H.-Y. Y., H.-Y. L., and H.-L. W. designed the experiments. T.-A. C., H.-Y. Y., H.-Y. L., Y.-T. C., and Z.-W. W. conducted the experiments. T.-A. C., H.-Y. Y., H.-Y. L., Y.-T. C., Z.-W. W., and H.-L. W. analyzed the data. H.-L. W. wrote the manuscript. All authors have given approval to the manuscript.

## Conflicts of interest

There are no conflicts to declare.

## Supplementary Material

SC-017-D5SC06015J-s001

## Data Availability

Additional experimental data supporting this article are provided in the supplementary information (SI). Reasonable requests for additional information can be made to the corresponding author. Supplementary information is available. See DOI: https://doi.org/10.1039/d5sc06015j.
