# Supplementary material for: Buried electrostatic modulation enables size-dependent reactivity in Pd-based nanocatalysts
Source: Chem Sci. 2025 Nov 26;17(3):1688–93. doi: 10.1039/d5sc06015j (PMC12670755; doi:10.1039/d5sc06015j)
Supplement: SC-017-D5SC06015J-s001 [file SC-017-D5SC06015J-s001.pdf]

Supplementary Information (SI) for Chemical Science.
This journal is © The Royal Society of Chemistry 2025

Supporting Information to

Buried Electrostatic Modulation Enables Size-Dependent Reactivity in Pd-Based Nanocatalysts

Tzu-An Chou‡, Hsiang-Yu Yu‡, Hui-Yun Lo‡, Yu-Ting Chen, Zhi-Wei Wang, Hsin-Lun Wu*

Department of Chemistry, National Cheng Kung University, Tainan, 70101, Taiwan

* *Corresponding author:* Hsin-Lun Wu

Email address: wuhsinlun@mail.ncku.edu.tw

Table of Contents

Experimental Section……………………………………………………………………..……..2
Synthesis of Au nanocubes (32, 52, and 72 nm)……………………………………….…. ….. 2
Synthesis of Pd nanocubes (35, 55, and 75 nm) ……………………………………….………... 3
Synthesis of thin-shell Au–Pd core–shell nanocubes (36, 56, and 76 nm) …………………..……. 3
Synthesis of thick-shell Au–Pd core–shell nanocubes (56, 76, and 96 nm) …….…………..……. 4
Catalysis for the 4-nitrophenol reduction reaction………………………..………………..…… 4
Catalysis for the Suzuki coupling reaction……………………...…………..……………..…..6
Figure S1…………………………………………………………………………………......8
Figure S2…………………………………………………………………………………......8
Figure S3…………………………………………………………………………………......9
Figure S4………………………………………………………………………………….....9
Figure S5…………………………………………………………………………………....10
Figure S6…………………………………………………………………………………....11
Figure S7…………………………………………………………………………………....12
Figure S8…………………………………………………………………………………....13

Experimental Section

Chemicals: Hydrogen tetrachloroaurate(III) trihydrate ($HAuCl_4 \cdot 3H_2O$, 99.99%, Alfa Aesar), palladium(II) chloride ($PdCl_2$, 99.9%, Sigma-Aldrich), cetyltrimethylammonium chloride (CTAC) ($C_{19}H_{42}ClN$, >95.0%, TCI), cetyltrimethylammonium bromide (CTAB) ($C_{19}H_{42}BrN$, ≥98.0%, Sigma-Aldrich), sodium borohydride ($NaBH_4$, ≥98.0%, Sigma-Aldrich), L-ascorbic acid ($C_6H_8O_6$, ≥99%, Sigma-Aldrich), sodium bromide (NaBr, 99%, J.T. Baker), potassium iodide (KI, ≥99.5%, Sigma-Aldrich), hydrochloric acid (HCl, ≥37%, Honeywell Fluka), 4-nitrophenol (4-NP) ($C_6H_5NO_3$, ≥99%, Sigma-Aldrich), iodobenzene (C_6H_5I, 98%, Alfa Aesar), phenylboronic acid ($C_6H_5B(OH)_2$, 95%, Sigma-Aldrich), potassium carbonate (K_2CO_3, 99%, Alfa Aesar).

Synthesis of Au nanocubes (32, 52, and 72 nm):[24]

Preparation of seed solution:

A 10.0 mL aqueous solution of 0.10 M CTAC (1.0 mmol) and 2.5×10^{-4} M $HAuCl_4$ (2.5 μmol) was prepared. Separately, 10.0 mL of 0.02 M $NaBH_4$ (0.2 mmol) was prepared in ice-cold water. Under stirring, 0.45 mL of the $NaBH_4$ solution was added to the $HAuCl_4$ solution, immediately forming a brown solution indicative of Au nanoparticle formation. The seed solution was aged at 30 ^oC for 30 min to decompose excess borohydride.

Preparation of growth solution:

Two vials, labeled A and B, were used to prepare growth solutions for Au nanocubes. To each vial, 0.32 g CTAC (1.0 mmol) and deionized water (9.550 mL for 32 nm, 9.600 mL for 52 nm, 9.620 mL for 72 nm) were added, yielding a final CTAC concentration of 0.10 M in 10 mL. The vials were maintained at 30 ^oC in a water bath. To each, 250 μL of 0.01 M $HAuCl_4$ (2.5 μmol), 10 μL of 0.01 M NaBr (0.1 μmol), and 90 μL of 0.04 M ascorbic acid (3.6 μmol) were added under stirring. The solution turned colorless, indicating reduction of Au^{3+} to Au^+.

To vial A, seed solution (100 μL for 32 nm, 50 μL for 52 nm, 30 μL for 72 nm) was added with stirring until the solution turned light pink (~5 s), indicating nucleation of Au nanoparticles. Subsequently, an aliquot from vial A (100 μL for 32 nm, 50 μL for 52 nm, 30 μL for 72 nm) was transferred to vial B and mixed thoroughly for ~10 s. The solution in vial B was left undisturbed for 30 min to allow particle growth. The resulting nanoparticles were centrifuged at 7500 rpm (32 nm), 6500 rpm (52 nm), or 3500 rpm (72 nm) for 10 min, and the supernatant was removed. The pellet was

resuspended in 10.0 mL of deionized water, centrifuged again under the same conditions, and the final product was stored in 1.0 mL of deionized water.

***Synthesis of Pd nanocubes (35, 55, and 75 nm)*:**[25,26]

Preparation of 0.01 M H_2PdCl_4 solution:

$PdCl_2$ (89 mg, 0.50 mmol) was dissolved in 50 mL of 0.02 M HCl aqueous solution. The solution was heated and ultrasonicated at 60 °C for 1 h.

Preparation of seed solution:

In a 50 mL vial, CTAB (91 mg, 0.25 mmol) and 20.0 mL of deionized water were stirred at 600 rpm and heated to 80 °C in a water bath. At 80 °C, 1.0 mL of 0.01 M H_2PdCl_4 (0.01 mmol) was added, and heating continued to 95 °C. Upon reaching 95 °C, the solution was stirred for 5 min, followed by addition of 160 μL of 0.1 M ascorbic acid (0.016 mmol). After stirring for 20 min, heating was stopped, and the solution was cooled to 60 °C in the water bath, yielding a brown solution containing ~17 nm Pd nanocube seeds for subsequent growth steps.

Preparation of growth solution:

In a 50 mL vial, CTAB (1.092 g, 3.0 mmol) and 30.0 mL of deionized water were mixed to dissolve. Subsequently, 750 μL of 0.01 M H_2PdCl_4 (0.0075 mmol) and 0.1 mM KI (0 μL for 35 nm, 50 μL for 55 nm and 75 nm) were added. The vial was placed in a 60 °C water bath and left undisturbed for 30 min to reach 60 °C.

To the growth solution, Pd nanocube seed solution (900 μL for 35 nm, 190 μL for 55 nm, 40 μL for 75 nm) and 300 μL of 0.1 M ascorbic acid (0.03 mmol) were added and mixed thoroughly. The solution was incubated at 60 °C in a water bath for 2.5 h. After reaction, the solution was centrifuged at 7500 rpm (35 nm), 6500 rpm (55 nm), or 3500 rpm (75 nm) for 10 min to remove the supernatant containing CTAB and ascorbic acid. The pellet was resuspended in 10.0 mL of deionized water, centrifuged again under the same conditions, yielding Pd nanocubes as the final product.

***Synthesis of thin-shell Au–Pd core–shell nanocubes (36, 56, and 76 nm)*:**[27]

CTAB (45 mg, 0.12 mmol) was dissolved in 9.0 mL of deionized water under magnetic stirring. Separately, a 1.0 mL of Au nanocube seed solution (32, 52, or 72 nm) was prepared by diluting the pre-synthesized Au nanocube stock solution with deionized water to match the concentration of a 70 μL aliquot that, when diluted to 870 μL, exhibited a UV-vis absorbance of 0.8 at the plasmonic peak. This seed solution was then added to the CTAB solution at room temperature, followed by either 125 μL (for 36 nm) or 100 μL (for 56 and 76 nm) of 0.01 M H_2PdCl_4 (0.00125 or 0.001 mmol,

respectively). The resulting mixture was stirred thoroughly and incubated in a water bath at 35 ^{o}C (36 nm), 43 ^{o}C (56 nm), or 40 ^{o}C (76 nm) at 230 rpm for 5 min. Then, 30 μL of 0.1 M ascorbic acid (0.003 mmol) was added, and the reaction was carried out under dark conditions with stirring for 90 min (36 nm), 60 min (56 nm), or 40 min (76 nm). The resulting solution was centrifuged at 7200 rpm (36 nm), 6500 rpm (56 nm), 3200 rpm (76 nm) for 10 min, and the supernatant containing CTAB and ascorbic acid was removed. The pellet was resuspended in 10.0 mL of deionized water, centrifuged again under the same conditions, yielding Au–Pd core–shell nanocubes with a 2 nm Pd shell and edge lengths of ~36 nm, 56 nm, or 76 nm.

For thick-shell Au–Pd core–shell nanocubes (56, 76, and 96 nm):

CTAB (125 mg, 0.34 mmol) was dissolved in 9.1 mL (for 56 nm) or 9.0 mL (for 76 and 96 nm) of deionized water under magnetic stirring. Separately, a 900 μL (for 56 nm) or 1000 μL (for 76 or 96 nm) of the pre-synthesized Au nanocube stock solution was added to bring the total volume to 10 mL. The Au seed concentration was prepared to match the concentration of a 70 μL aliquot that, when diluted to 800 μL, exhibited a UV-vis absorbance of 0.8 at the plasmonic peak. This mixture was followed by the addition of 350 μL (for 56 nm), 430 μL (for 76 nm), or 400 μL (for 96 nm) of 0.01 M H_2PdCl_4 (0.0035 mmol, 0.0043 mmol, or 0.0040 mmol, respectively). The resulting mixture was stirred thoroughly and incubated in a water bath at 40 ^{o}C (56 nm), 43 ^{o}C (76 nm), or 42 ^{o}C (96 nm) at 230 rpm for 5 min. Then, 280 μL (56 nm), 200 μL (76 nm), or 50 μL (96 nm) of 0.1 M ascorbic acid (0.028 mmol, 0.020 mmol, or 0.005 mmol, respectively) was added, and the reaction was carried out under dark conditions with stirring for 90 min (56 and 96 nm) or 60 min (76 nm). The resulting solution was centrifuged at 6500 rpm (56 nm), 3500 rpm (76 nm), or 2500 rpm (96 nm) for 10 min, and the supernatant containing CTAB and ascorbic acid was removed. The pellet was resuspended in 10.0 mL of deionized water, centrifuged again under the same conditions, yielding Au–Pd core–shell nanocubes with a 12 nm Pd shell and edge lengths of ~56 nm, 76 nm, or 96 nm.

***Catalysis for the 4-nitrophenol reduction reaction*:**

In a 20 mL vial, 1.6 mL of freshly prepared 0.1 M $NaBH_4$ (0.16 mmol) and 0.16 mL of 0.01 M 4-nitrophenol (0.0016 mmol) aqueous solutions were subsequentially added. The vial was placed inside a beaker in an ultrasonic bath under dark conditions to maintain a stable temperature. Subsequentially, 6.24 mL of an aqueous solution containing Au, Pd, or Au–Pd nanocubes (surface area concentration: 0.3 cm^2/mL) was added, bringing the total reaction volume to 8.00 mL. The equivalent nanocube masses used to achieve this surface area concentration were: 0.0030 mg/mL (32 nm Au), 0.0049 mg/mL (52 nm Au), 0.0068 mg/mL (72 nm Au), 0.0021 mg/mL (35 nm Pd), 0.0033 mg/mL (55 nm

Pd), 0.0045 mg/mL (75 nm Pd), 0.0031 mg/mL (36 nm thin Au–Pd), 0.0050 mg/mL (56 nm thin Au–Pd), 0.0069 mg/mL (76 nm thin Au–Pd), 0.0037 mg/mL (56 nm thick Au–Pd), 0.0054 mg/mL (76 nm thick Au–Pd), 0.0072 mg/mL (96 nm thick Au–Pd). The reaction was initiated upon addition of the nanocube solution. At 3-minute intervals, 1.0 mL of the reaction mixture was withdrawn and transferred into a quartz cuvette containing 2.0 mL of deionized water for UV-Vis analysis. The absorbance at 400 nm (A) was recorded seven times. The apparent first-order rate constant (k, min^{-1}) was determined by plotting $\ln(A/A_0)$ versus time, where A_0 represents the initial absorbance.

Calculation of the surface area of 0.3 cm^2 for the nanocubes:

Taking 32 nm Au as an example: The edge length of the Au nanocubes is 32 ± 1.3 nm. The volume of a single Au nanocubes at 32 nm is 3.28×10^{-17} cm^3. The density of Au is 19.3 g/cm^3. Given that 0.0030 mg of 32 nm Au nanocubes was used in the 4-nitrophenol reduction reaction, the number of Au nanocubes is calculated as $(3.0 \times 10^{-6}$ g) / (19.3 g/cm^3 × 3.28×10^{-17} cm^3) = 4.74×10^{9}. The surface area of a single Au nanocube is 6.14×10^{-11} cm^2. Therefore, the total surface area of the Au nanocubes used is $(4.74 \times 10^{9}) \times (6.14 \times 10^{-11}$ cm^2) = 0.3 cm^2.

Taking 35 nm Pd as an example: The edge length of the Pd nanocubes is 35 ± 1.2 nm. The volume of a single Pd nanocubes at 35 nm is 4.29×10^{-17} cm^3. The density of Pd is 12.02 g/cm^3. Given that 0.0021 mg of 35 nm Pd nanocubes was used in the 4-nitrophenol reduction reaction, the number of Pd nanocubes is calculated as $(2.1 \times 10^{-6}$ g) / (12.02 g/cm^3 × 4.29×10^{-17} cm^3) = 4.07×10^{9}. The surface area of a single Pd nanocube is 7.35×10^{-11} cm^2. Therefore, the total surface area of the Pd nanocubes used is $(4.07 \times 10^{9}) \times (7.35 \times 10^{-11}$ cm^2) = 0.3 cm^2.

Taking 36 nm thin-shell Au–Pd (core: 32 nm) as an example: The edge length of the Au–Pd nanocubes is 36 ± 1.2 nm. The volume of a single Au–Pd nanocubes at 36 nm is 4.69×10^{-17} cm^3. The densities of Au and Pd are 19.3 g/cm^3 and 12.02 g/cm^3, respectively. Given that 0.0031 mg of 36 nm Au-Pd nanocubes was used in the 4-nitrophenol reduction reaction, the number of Au–Pd nanocubes is calculated as $(3.1 \times 10^{-6}$ g) / [19.3 g/cm^3 × 3.28×10^{-17} + 12.02 g/cm^3 × $(4.69 \times 10^{-17} - 3.28 \times 10^{-17})$ cm^3] = 3.86×10^{9}. The surface area of a single Au–Pd nanocube is 7.78×10^{-11} cm^2. Therefore, the total surface area of the Au nanocubes used is $(3.86 \times 10^{9}) \times (7.78 \times 10^{-11}$ cm^2) = 0.3 cm^2.

Taking 56 nm thick-shell Au–Pd (core: 32 nm) as an example: The edge length of the Au–Pd nanocubes is 56 ± 1.2 nm. The volume of a single Au–Pd nanocubes at 56 nm is 1.77×10^{-16} cm^3. The densities of Au and Pd are 19.3 g/cm^3 and 12.02 g/cm^3, respectively. Given that 0.0037 mg of 56 nm Au–Pd nanocubes was used in the 4-nitrophenol reduction reaction, the number of Au–Pd nanocubes is calculated as $(3.7 \times 10^{-6}$ g) / [19.3 g/cm^3 × 3.28×10^{-17} + 12.02 g/cm^3 × $(1.77 \times 10^{-16} - 3.28 \times 10^{-17})$

cm^3] = 1.56 × 10^9. The surface area of a single Au–Pd nanocube is 1.88 × 10^{-10} cm^2. Therefore, the total surface area of the Au nanocubes used is (1.56 × 10^9) × (1.88 × 10^{-10} cm^2) = 0.3 cm^2.

***Catalysis for the Suzuki coupling reaction*:**

Phenylboronic acid (36.6 mg, 0.30 mmol), K_2CO_3 (69.0 mg, 0.50 mmol), and CTAB (36.0 mg, 0.10 mmol) were dissolved in a mixture of 0.5 mL ethanol and 0.5 mL deionized water, and stirred at 800 rpm for 1h to ensure homogeneity. The solution was transferred to a quartz cuvette for catalysis, followed by the addition of iodobenzene (17 μL, 0.15 mmol). After stirring for 5 min at 800 rpm, the reaction was initiated by adding the nanocube catalyst with a total surface area of 10 cm^2 under dark conditions. The corresponding masses of nanocubes used to achieve this surface area were: 0.0714 mg (35 nm Pd), 0.1104 mg (55 nm Pd), 0.1482 mg (75 nm Pd), 0.1025 mg (36 nm Au–Pd), 0.1669 mg (56 nm Au–Pd), 0.2314 mg (76 nm Au–Pd). After 1h, the reaction mixture was centrifuged at 10,000 rpm for 10 min. The precipitate was redissolved in 2.0 mL ethanol, centrifuged again, and the combined supernatants were adjusted to a total volume of 5.0 mL. The solution was sonicated for 5 min to ensure homogeneity, and a 50 μL aliquot was then diluted with 7.95 mL ethanol for GC-MS analysis.

Calculation of the total number of surface Pd atoms on Pd and Au–Pd nanocubes used in the Suzuki coupling reaction for turnover frequency (TOF) determination:

Taking 35 nm Pd as an example: The edge length of the Pd nanocubes is 35 nm. The volume of a single Pd nanocube is 4.3 × 10^{-17} cm^3. The density of Pd is 12.02 g/cm^3. Because 0.0714 mg of Pd nanocubes was used in the Suzuki coupling reaction, the number of nanocubes (N_{Pd}) is (7.14 × 10^{-5} g)/(12.02 g/cm^3 × 4.3 × 10^{-17} cm^3) = 1.4 × 10^{11}. The surface area of a single Pd nanocube is (35 nm)^2 × 6 = 7.35 × 10^3 nm^2. Pd exists in a face-centered cubic phase with a lattice constant of 0.389 nm. Each two-dimensional unit cell on the (200) facet contains two Pd atoms. The area of a unit cell is (0.389 nm)^2 = 0.151 nm^2. The number of surface Pd atoms on a single Pd nanocube is (7.35 × 10^3 nm^2)/(0.151 nm^2) × 2 = 9.74 × 10^4. The total number of surface Pd atoms on the Pd nanocubes in the Suzuki coupling reaction is (9.74 × 10^4) × (1.4 × 10^{11}) = 1.3 × 10^{16}.

Taking 36 nm thin-shell Au–Pd (core: 32 nm) as an example: The edge length of the Au–Pd nanocubes is 36 nm. The volume of a single Au nanocube is 3.3 × 10^{-17} cm^3. The volume of a single Au–Pd nanocube is 4.7 × 10^{-17} cm^3. The densities of Au and Pd are 19.3 g/cm^3 and 12.02 g/cm^3, respectively. Because 0.1025 mg of Au–Pd nanocubes was used in the Suzuki coupling reaction, the number of

nanocubes ($N_{Au–Pd}$) is (1.025×10^{-4} g)/[19.3 g/cm^3 × 3.3×10^{-17} cm^3 + 12.02 g/cm^3 × (4.7×10^{-17} – 3.3×10^{-17} cm^3)] = 1.3×10^{11}. The surface area of a single Au–Pd nanocube is (36 nm)^2 × 6 = 7.78×10^3 nm^2. Pd exists in a face-centered cubic phase with a lattice constant of 0.389 nm. Each two-dimensional unit cell on the (200) facet contains two Pd atoms. The area of a unit cell is (0.389 nm)^2 = 0.151 nm^2. The number of surface Pd atoms on a single Au–Pd nanocube is (7.78×10^3 nm^2)/(0.151 nm^2) × 2 = 1.03×10^5. The total number of surface Pd atoms on the Au–Pd nanocubes in the Suzuki coupling reaction is (1.03×10^5) × (1.3×10^{11}) = 1.3×10^{16}.

Taking 56 nm thick-shell Au–Pd (core: 32 nm) as an example: The edge length of the Au–Pd nanocubes is 56 nm. The volume of a single Au nanocube is 3.3×10^{-17} cm^3. The volume of a single Au–Pd nanocube is 1.8×10^{-16} cm^3. The densities of Au and Pd are 19.3 g/cm^3 and 12.02 g/cm^3, respectively. Because 0.1260 mg of Au–Pd nanocubes was used in the Suzuki coupling reaction, the number of nanocubes ($N_{Au–Pd}$) is (1.260×10^{-4} g)/[19.3 g/cm^3 × 3.3×10^{-17} cm^3 + 12.02 g/cm^3 × (1.8×10^{-16} – 3.3×10^{-17} cm^3)] = 5.3×10^{10}. The surface area of a single Au–Pd nanocube is (36 nm)^2 × 6 = 1.88×10^4 nm^2. Pd exists in a face-centered cubic phase with a lattice constant of 0.389 nm. Each two-dimensional unit cell on the (200) facet contains two Pd atoms. The area of a unit cell is (0.389 nm)^2 = 0.151 nm^2. The number of surface Pd atoms on a single Au–Pd nanocube is (1.88×10^4 nm^2)/(0.151 nm^2) × 2 = 2.49×10^5. The total number of surface Pd atoms on the Au–Pd nanocubes in the Suzuki coupling reaction is (2.49×10^5) × (5.3×10^{10}) = 1.3×10^{16}.

Characterization: Transmission electron microscopy (TEM) was conducted using a JEM-2100F microscope (JEOL) operating at 100 kV. Scanning electron microscope (SEM) was performed with a Hitachi SU8000 system. Powder X-ray diffraction (XRD) patterns were collected on a Bruker D2 Phaser using Cu Kα radiation. UV-vis spectra were recorded with a Shimadzu UV-2600 spectrophotometer. Gas chromatography-mass spectrometry (GC-MS) analysis was carried out using a Shimadzu GCMS-QP2010SE instrument.

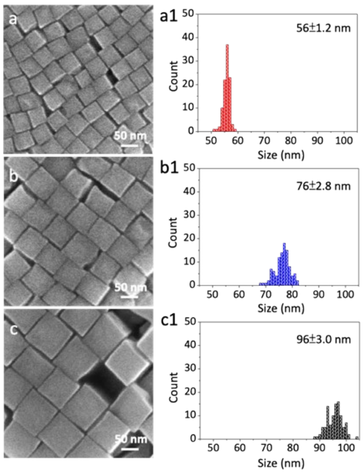

Figure S1. SEM images of (a–c) thick-shell (~12 nm) Au–Pd core–shell nanocubes, with corresponding size distributions shown in (a1–c1). These thick-shell nanocubes serve as a control to assess the influence of shell thickness on electrostatic responsiveness and to exclude effects from lattice strain.

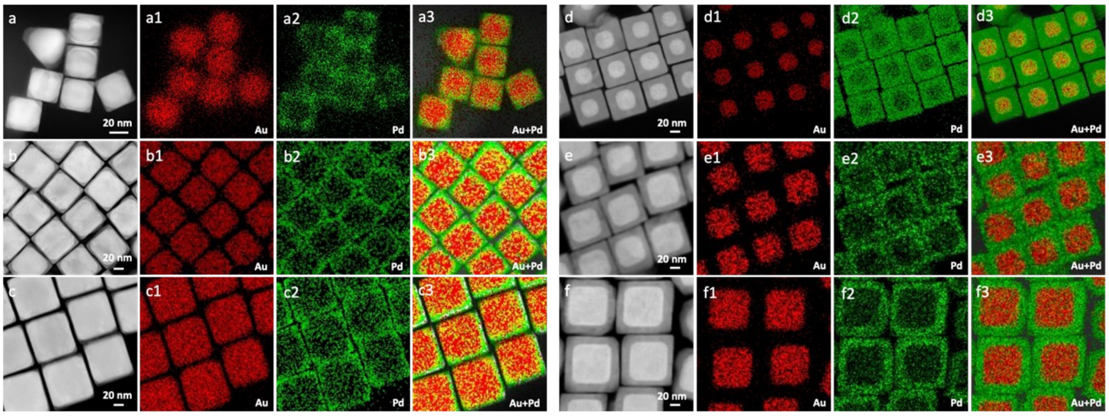

Figure S2. HAADF-STEM images and corresponding EDS elemental mapping of Au–Pd core–shell nanocubes. Thin-shell (~2 nm) samples with edge lengths of (a–a3) 36 nm, (b–b3) 56 nm, and (c–c3) 76 nm, and thick-shell (~12 nm) samples with edge lengths of (d–d3) 56 nm, (e–e3) 76 nm, and (f–f3) 96 nm are shown. In all cases, Pd (green) forms a uniform shell around the Au core (red), confirming the core–shell structure.

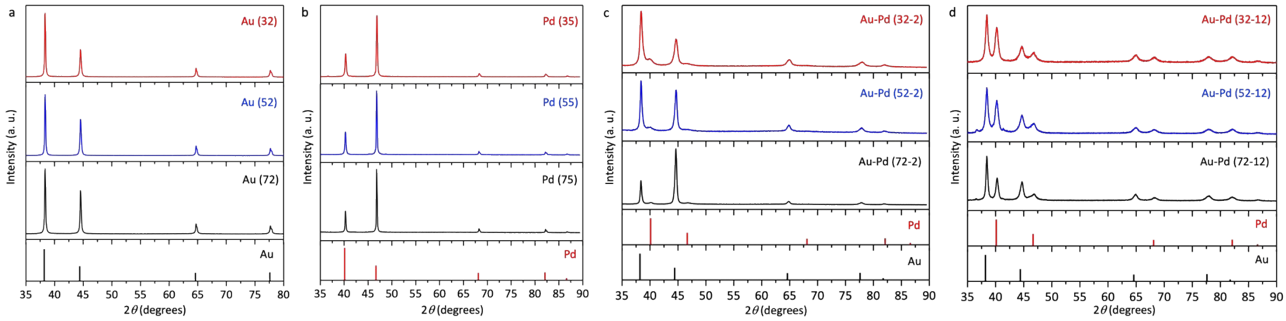

Figure S3. XRD patterns of (a) Au nanocubes, (b) Pd nanocubes, (c) thin-shell (~2 nm) Au–Pd core–shell nanocubes, and (d) thick-shell (~12 nm) Au–Pd core–shell nanocubes. The diffraction peaks confirm the face-centered cubic structures of Au and Pd, with the Au–Pd core–shell pattern displaying characteristic features of both metals, indicating successful formation of the core–shell structure for both thin- and thick-shell samples.

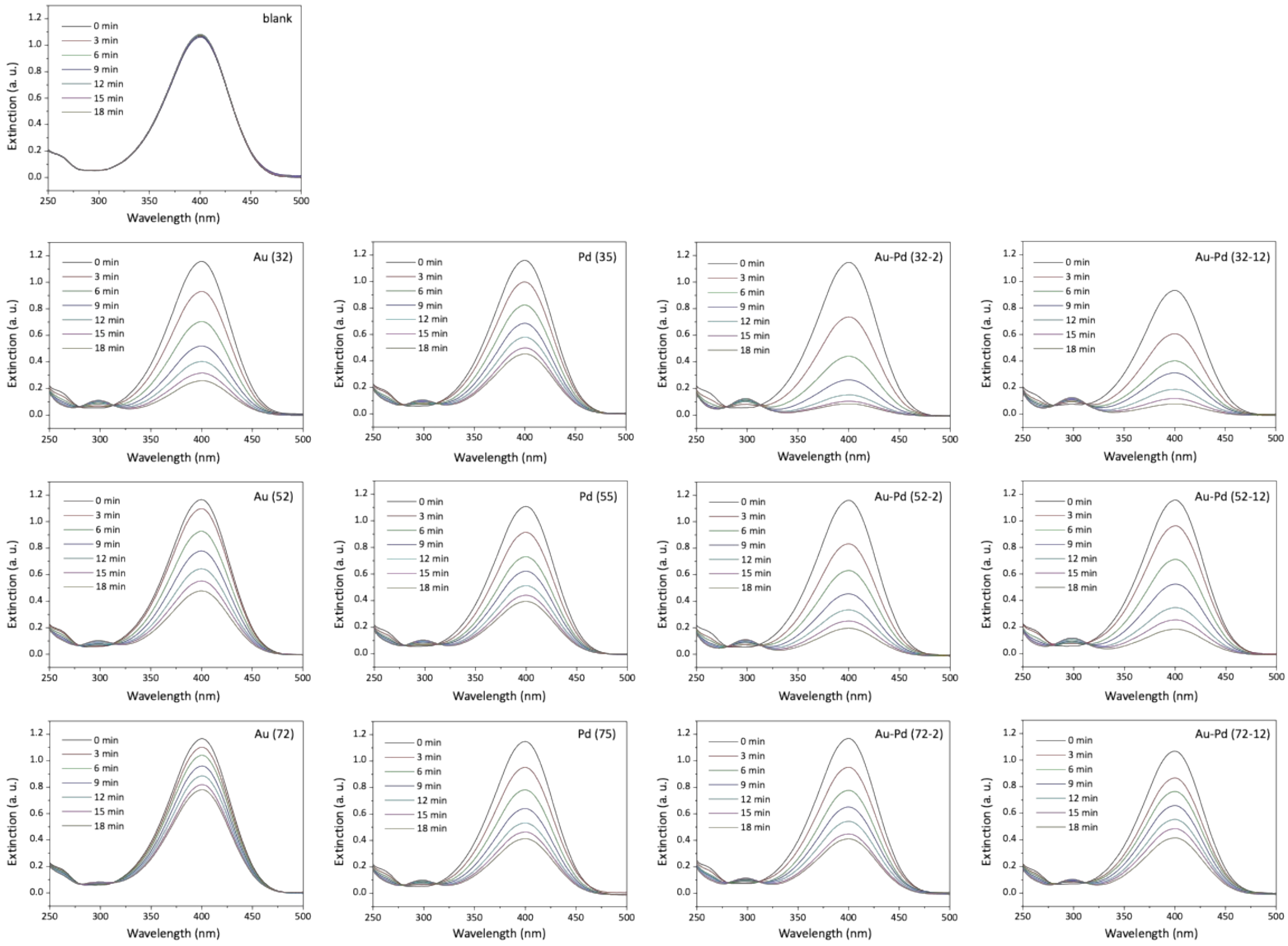

Figure S4. UV-vis spectra of 4-nitophenol reduction catalyzed by Au, Pd, and Au–Pd core–shell nanocubes of varying sizes under dark conditions. The figure includes spectra for monometallic Au

and Pd nanocubes, as well as thin-shell (~2 nm) and thick-shell (~12 nm) Au–Pd core–shell nanocubes. All spectra show a gradual decrease in the absorption peak at ~400 nm (4-nitrophenol) and a concurrent increase at ~300 nm (4-aminophenol), indicating catalytic conversion. These spectra provide the basis for calculating the reaction kinetics presented in Figure 3.

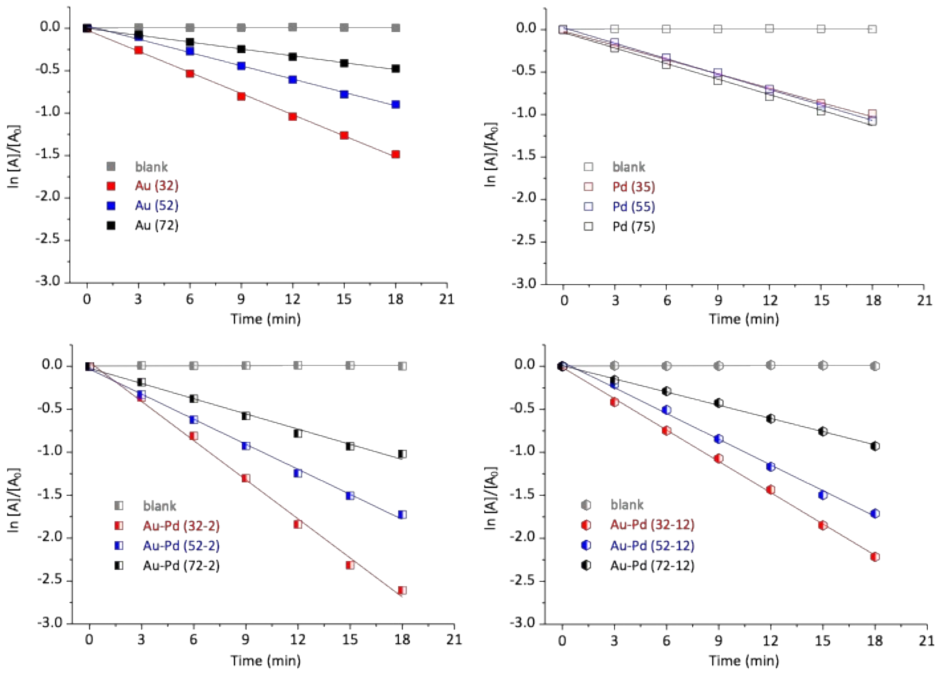

Figure S5. Plots of $\ln(A/A_0)$ versus time for the 4-nitrophenol reduction catalyzed by Au, Pd, and Au–Pd core–shell nanocubes of various sizes under dark conditions. The figure includes kinetic data for monometallic Au and Pd nanocubes, as well as thin-shell (~2 nm) and thick-shell (~12 nm) Au–Pd core–shell nanocubes. These plots are used to determine the apparent first-order rate constants presented in Figure 3.

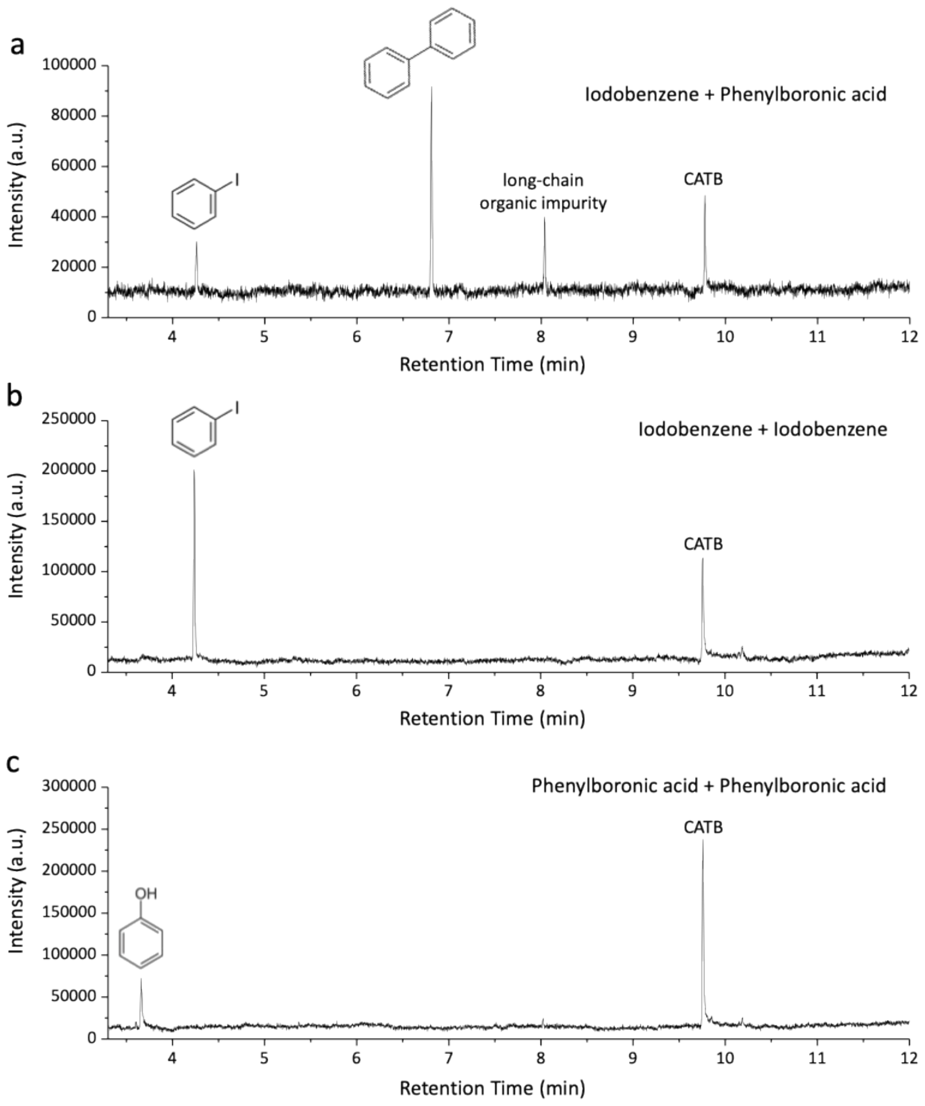

Figure S6. GC–MS chromatograms of the Suzuki coupling reaction and related control experiments. (a) Reaction catalyzed by Au–Pd core–shell nanocubes, showing the desired product biphenyl (6.8 min) and unreacted iodobenzene (4.3 min). The intense peak at 9.8 min corresponds to residual CTAB surfactant, while a minor peak at 8.0 min arises from a long-chain organic impurity associated with the CTAB system and is not a coupling product. No characteristic peaks of typical Suzuki side-products (e.g., homo-coupled or dehalogenated species) are observed, confirming nearly 100% selectivity toward biphenyl. (b) Control experiment using only iodobenzene (Ph–I) under identical reaction conditions, showing no formation of biphenyl, indicating that homo-coupling does not occur in the absence of phenylboronic acid. (c) Control experiment using only phenylboronic acid (Ph–

B(OH)₂) under identical conditions, showing no biphenyl formation, further confirming that the observed product originates exclusively from the cross-coupling pathway.

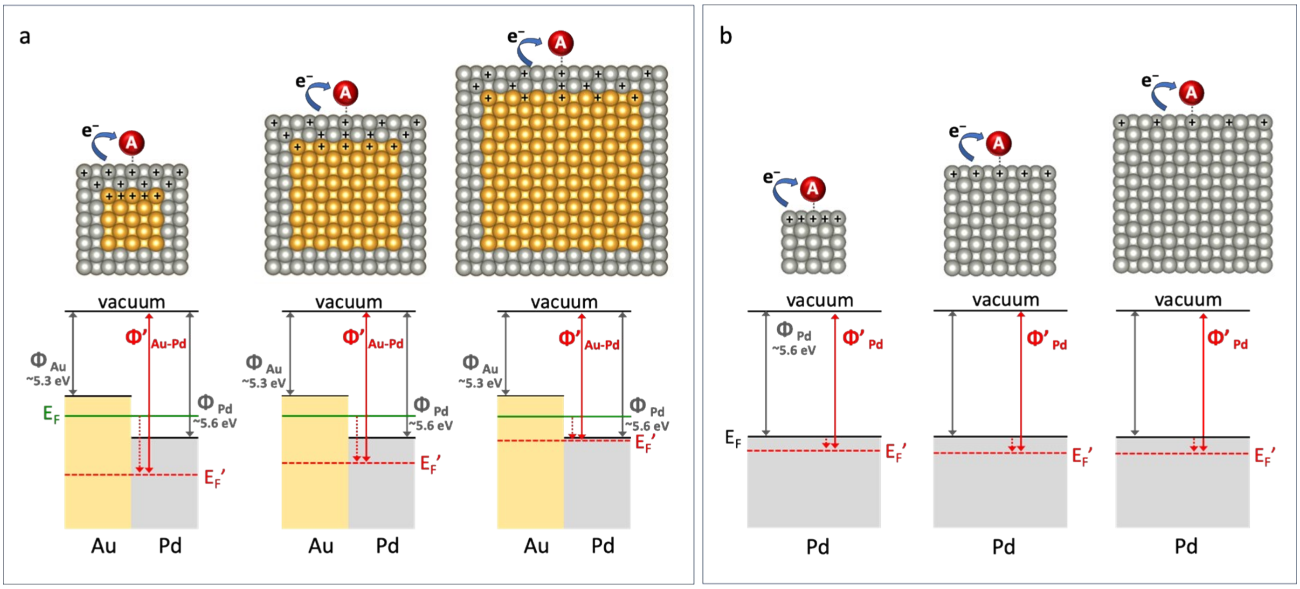

Figure S7. Schematic illustration of E_F shifts under transient positive charge accumulation. (a) Au–Pd core–shell nanocubes: Electrons initially flow from Au (5.3 eV) to Pd (5.6 eV), establishing an equilibrium E_F (green line) representing the ligand effect. Under transient positive charge accumulation, the buried Au core amplified E_F lowering, overriding the initial equilibrium, especially in smaller particles. (b) Pd nanocubes: E_F shifts remain size-independent, reflecting Pd's low electrostatic responsiveness even under transient positive surface charge.

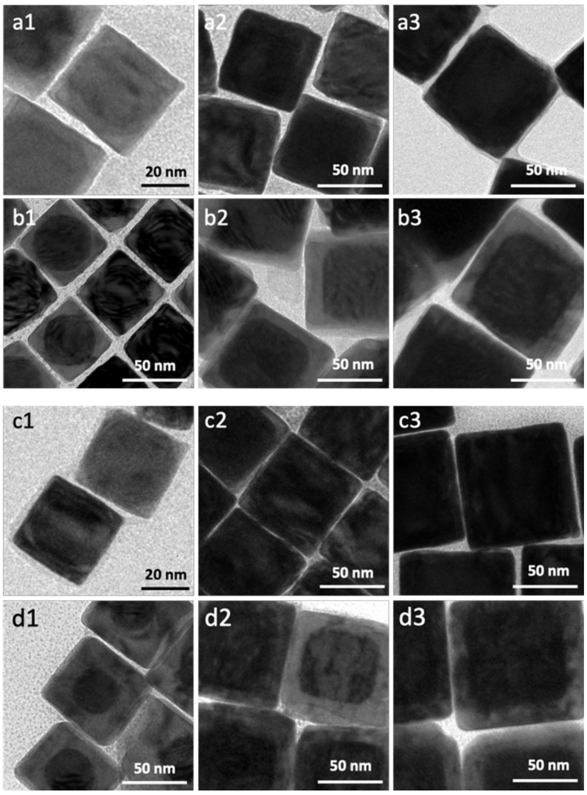

Figure S8. TEM images of Au–Pd core–shell nanocubes after catalytic reactions. (a1–a3) Thin-shell and (b1–b3) thick-shell Au–Pd nanocubes after 4-nitrophenol reduction, and (c1–c3) thin-shell and (d1–d3) thick-shell Au–Pd nanocubes after Suzuki coupling reaction. All samples preserved their cubic morphology and distinct core–shell structures, confirming the structural stability of both thin- and thick-shell Au–Pd nanocubes under the applied catalytic conditions, without detectable morphology distortion, alloying, or metal leaching.
